# Vector Beam Polarization State Spectrum Analyzer

**DOI:** 10.1038/s41598-017-02328-5

**Published:** 2017-05-22

**Authors:** Ignacio Moreno, Jeffrey A. Davis, Katherine Badham, María M. Sánchez-López, Joseph E. Holland, Don M. Cottrell

**Affiliations:** 1 Departamento de Ciencia de Materiales, Óptica y Tecnología Electrónica, Universidad Miguel Hernández de Elche, 03202 Elche, Spain; 2 Department of Physics, San Diego State University, California, 92182-1233 USA; 3 Instituto de Bioingeniería y Departamento de Física y Arquitectura de Computadores, Universidad Miguel Hernández, 03202 Elche, Spain

## Abstract

We present a proof of concept for a vector beam polarization state spectrum analyzer based on the combination of a polarization diffraction grating (PDG) and an encoded harmonic *q*-plate grating (QPG). As a result, a two-dimensional polarization diffraction grating is formed that generates six different *q*-plate channels with topological charges from −3 to +3 in the horizontal direction, and each is split in the vertical direction into the six polarization channels at the cardinal points of the corresponding higher-order Poincaré sphere. Consequently, 36 different channels are generated in parallel. This special polarization diffractive element is experimentally demonstrated using a single phase-only spatial light modulator in a reflective optical architecture. Finally, we show that this system can be used as a vector beam polarization state spectrum analyzer, where both the topological charge and the state of polarization of an input vector beam can be simultaneously determined in a single experiment. We expect that these results would be useful for applications in optical communications.

## Introduction

In recent years there has been a very intense research activity in the development of communication systems based on multiplexing of vortex beams having angular orbital momentum (OAM)^1^. They have been demonstrated in fibers^2^, and in free space^3, 4^. The technique can be generalized to the use of more general Laguerre-Gaussian (LG) beams^5, 6^. Free-space communications over 143 km^7^, and rates up to 500 Gb/s have been achieved^8^.

In all cases, a multiplexer/demultiplexer element is required to generate and detect the different channels used in the communication system^9^. One classical element that allows the parallel generation and detection of vortex beams is the fork grating or vortex grating^10^, where a phase spiral pattern is added to the linear phase. When the phase profile of this fork grating is modified, multiple harmonic components arise in the grating, allowing the parallel generation of different vortex beams on different diffraction orders^11^. The same type of grating, when illuminated with a vortex beam, presents a bright diffraction order (delta function) at the diffraction order with the charge that compensates for the charge in the incoming beam, therefore acting as a vortex beam detector. The combination of the vortex grating with a Dammann grating allows generating vortex diffraction orders with the same intensity^12^. Therefore, this Dammann vortex grating can be used as a vortex spectrum analyzer, where the different weights of vortex charges can be measured by the relative intensities at the center of the corresponding diffraction order^4, 13^.

The application of the optimal efficient phase profile for fan-out diffractive elements^14^ allowed the design of these vortex sensing phase gratings with arbitrary target diffraction orders, with arbitrary intensity^15^ and with arbitrary relative phase^16^. The same approach can be applied to design two different phase gratings to be applied to two orthogonal states of polarizations, in order to produce a polarization diffraction grating (PDG) where the state of polarization can be defined at will at each diffraction order^17^.

In attempts to increase the information content in optical communication systems, researchers have been investigating vector beams^18^. These beams are characterized by a two dimensional polarization state pattern that depends on both the charge (OAM) of the generating device and the polarization state encoded onto the beam. Typically, these applications require projections of the vortex beam onto one of the 6 cardinal points on the appropriate Poincaré sphere. Among the most popular techniques for generating these vector beams are the *q*-plate devices^19^. These are linear retarders with a half-wave retardance where the optical axis continuously follows the azimuth angle *θ* as *qθ*,where the *q*-value gives the vector beam charge as 2*q*. Q-plates have been studied in different communications schemes^20, 21^.

Q-plates can be fabricated with structured metamaterials^22^ (then also named as *s*-plates), with elements based on geometric phase holograms^23^, or with liquid-crystal materials^24, 25^, these last showing the possibility to tune the retardance and be operative at different wavelengths. Alternatively, they can be encoded using spatial light modulators (SLM)^26, 27^. The flexibility provided by SLMs allows the generation of arbitrary *q*-plates.

In this work, we demonstrate a vector beam polarization state spectrum analyzer with the capability to detect both the charge of the *q*-plate used for its generation, as well as the specific polarization state that has been encoded. This extends the vortex beam spectrum analyzer^11^ to vectorial beams, and is much more complex because of the greater amount of information that must be encoded. This extension requires the combined use of various powerful techniques that have been developed previously.

In our approach, we create a combined diffractive element that consists of two orthogonal grating structures. Using our approach for generating *q*-plates^27^, we make a vector beam diffraction grating that simultaneously creates 6 different vector beams with different charges in the horizontal diffracted orders. In addition, we combine this with a previously reported polarization diffraction grating^17^ in the vertical diffracted orders where each order has a different polarization state on the zero-order Poincaré sphere. As a result, we are able to generate, in a single shot, 6 × 6 different vector beams where the horizontal order shows different topological charge, while the vertical order shows different states of polarization. This generalizes previous approaches to produce vector beams in parallel^28, 29^.

Let us remark that the same system, acting in a reverse sense, can be used also as a vector beam analyzer, useful to detect the charge and polarization of an incoming arbitrary vector beam. Experimental results are given demonstrating this capability.

## Methods and Techniques

Next we summarize the different experimental methods required to achieve the vector beam diffraction grating analyzer.

### Experimental system to encode *q*-plates

The experimental system is inspired in a previous decomposition of the *q*-plate Jones matrix^27^. A general *q*-plate^10^ can be described with the following Jones matrix:

1 $${{\bf{M}}}_{q}=(\begin{array}{cc}\cos (2q\theta ) & \sin (2q\theta )\\ \sin (2q\theta ) & -\cos (2q\theta )\end{array})$$

where *θ* is the azimuth angle. This matrix can be decomposed as the combination of a polarization rotator and a half-wave retarder as:

2 $${{\bf{M}}}_{q}={\bf{H}}{\bf{W}}{\bf{P}}\cdot {\bf{R}}(2q\theta )$$

where **HWP** = diag(+1, −1), and **R**(*α*) is the rotation matrix of angle *α*. We follow the notation in ref. 30.

This above relation can be further decomposed by noting that the polarization rotator can be generated from a linear retarder placed in between two quarter-wave plates oriented at ±45° ^31^, therefore leading to:

3 $${{\bf{M}}}_{q}={\bf{H}}{\bf{W}}{\bf{P}}\cdot {\bf{Q}}{\bf{W}}{\bf{P}}(+45^\circ)\cdot {\bf{W}}(\varphi )\cdot {\bf{Q}}{\bf{W}}{\bf{P}}(-45^\circ)$$

Here the matrix **W**(*ϕ*) corresponds to a linear retarder with an azimuthally varying retardance *ϕ* = 2ℓ*θ*:

4 $${\bf{W}}(\varphi )=(\begin{array}{cc}\exp (+i\ell \theta ) & 0\\ 0 & \exp (-i\ell \theta )\end{array})$$

The matrices for the quarter-wave retarders are **QWP**(*α*) = **R**(−*α*)·**QWP**(0)·**R**(+*α*) and **QWP**(0) = diag(1, −*i*). With this choice for the retardance, the system of Eq. (3) will produce a *q*-plate where the value of *q* is related to the value of ℓ by ℓ = 2*q*.

Next, we discuss the experimental setup. In our optical system, light from an argon laser at a wavelength of 514 nm goes through a spatial filter and an initial polarizing optical system. This polarization system consists of a linear polarizer (LP) and a quarter-wave plate (QWP) and is capable of producing any polarization state. The beam is collimated by a lens with a 52 cm focal length and passes through a circular aperture whose diameter is equivalent to 240 pixels of our spatial light modulator.

**Figure 1:**
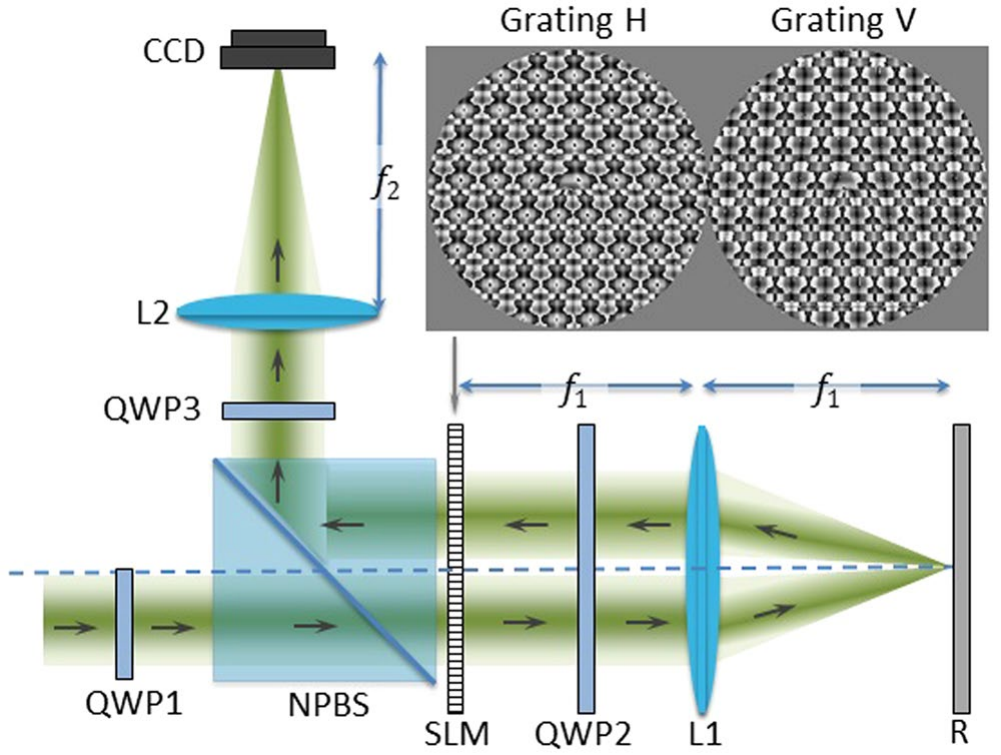
Experimental system. SLM: parallel-aligned spatial light modulator. QWP: quarter-wave plate. L: converging lens. NPBS: non-polarizing beam-splitter. R: mirror. The SLM displays two phase-only gratings with embedded spiral phases (shown in the insert) that are encoded on two orthogonal states of polarization.

The most important part of the experimental system is shown in Fig. 1. As mentioned earlier, the central matrix in Eq. (3) requires an optical system capable to encode two independent phase patterns on two orthogonal linear states of polarization. Since liquid crystal displays only modulates the polarization component parallel to the liquid crystal director, we use a double-pass architecture as indicated in Fig. 1. The light first passes through a non-polarizing beam-splitter onto a phase-only SLM.

The basis for the PDG is a polarization rotation system reported earlier^31^ where a linear retarder SLM is located between two orthogonal QWPs (QWP1 and QWP3 in Fig. 1) whose optical axes are oriented at ±45 degrees relative to the vertical axis. That work showed that the output polarization state is rotated by an angle of *θ* = *ϕ*/2 where *ϕ* is the retardance encoded onto the SLM. In this current work, however, we are required to encode independent phase patterns onto two orthogonal polarizations. This can be achieved either using two different SLMs^28^, or using one single SLM where the screen is divided in two halves^26^. Here we use this last approach, in a previously demonstrated system^32^.

The key component is a transmissive zero-twist liquid crystal SLM having 640 × 480 pixels with a 42 micron pixel size. Each pixel acts as an electrically controlled wave-plate with a phase shift that exceeds 2*π* radians when illuminated with an Ar laser at 514.5 nm^31^. The LC director is vertically oriented in the laboratory framework. The screen of the SLM is divided in two halves, where two different phase patterns are addressed. The initial beam illuminates only the pattern on the left, which is encoded onto the vertical linear polarization. The horizontal component is unaffected. Then, by means of a lens having a focal length of 37 cm (L3) and a mirror (R), the beam is reflected back to the right part of the SLM. The initially vertical and horizontal linearly polarized components are reversed by the insertion of a quarter-wave plate (QWP2), oriented at 45° with respect to the LC director axis. Now the horizontal linear polarization component, which was not affected by the SLM in the initial passage, becomes vertically polarized and will be modulated by the second phase pattern encoded on the right side of the LCD.

The output beam, with the different phase patterns on each polarization axis, reflects from the non-polarizing beam-splitter, passes through a lens having a focal length of 37 cm and forms the desired vector beams. Finally, we add a polarization analyzer, composed by another QWP and a linear polarizer, which is placed in front of the WinCamD camera that is located at the image plane.

This system was demonstrated in ref. 27 to generate *q*-plates, with arbitrary integer and fractional *q* values. This is achieved by encoding spiral phases with opposite sense in each side of the SLM. However, as we show next, the same system can be used to combine the encoded *q*-plates with optimal phase gratings to create arrays of vector beams and a vector beam spectrum analyzer.

### Encoding harmonic *q*-plate gratings

Since we are interested in generating vector beams with different charges in a single shot, we can use the spiral phase embedded into a diffraction grating, to create a fork grating. This way, each diffraction order *k* generates a vortex phase with topological charge *k*ℓ, where ℓ denotes the charge of the embedded spiral phase in Eq. (4), and the grating can be used as a parallel generator/detector of vortex beams^11^.

Then, a proper look-up table can be applied following the procedure initiated by Romero and Dickey^14^, and extensively discussed in previous works^15, 16^, to equalize the energy on the different target diffraction orders. Following this procedure, a set *T* of target diffraction orders are selected in the grating, each described by a linear phase term exp(*i*2*πkx*/*D*), where *k* is the integer order that denotes the order, *D* is the period of the grating, and where we selected the grating oriented in the *x*-coordinate.

Then, each harmonic component is controlled by a different complex amplitude value, ${\mu }_{k}{e}^{i{\alpha }_{k}}$. The two parameters (modulus and phase, *μ*_*k*_, *α*_*k*_) for each target order are numerically calculated to provide a desired constraint between the complex amplitudes *G*_*k*_ = |*G*_*k*_|exp(*iβ*_*k*_) of each diffraction orders generated by the grating, so the finally designed phase-only grating can be written as:

5 $$\exp (i\phi (x))=\frac{\sum _{k\in T}{\mu }_{k}{e}^{i{\alpha }_{k}}{e}^{i2\pi kx/D}}{|\sum _{k\in T}{\mu }_{k}{e}^{i{\alpha }_{k}}{e}^{i2\pi kx/D}|}=\sum _{k=-\infty }^{+\infty }{G}_{k}{e}^{i2\pi kx/D}.$$

We followed this procedure to design a grating, diffracting in the horizontal direction, that generates six equally intense diffraction orders *k* = ± 1, ±2, ±3. The total diffraction efficiency of the phase grating design exceeds 90%. We note that our grating designs provide diffraction efficiencies using this procedure are much higher than others have obtained^28^. A spiral phase of charge ℓ is embedded in the grating before applying the phase LUT profile, so the diffraction order *k* generates a harmonic vortex beam with charge *k*ℓ^11^.

This grating design can be used to generate harmonic *q*-plates simply by selecting the same grating for each polarization component in the system in Fig. 1, but selecting the embedded spiral phase with opposite charge in each polarization. Then, the Jones matrix **W** for the linear retarder component in Eq. (4) can be written as:

6 $${\bf{W}}=(\begin{array}{cc}\sum _{k}{G}_{k}{e}^{ik\ell \theta }{e}^{i2\pi kx/D} & 0\\ 0 & \sum _{k}{G}_{k}{e}^{-ik\ell \theta }{e}^{i2\pi kx/D}\end{array})=\sum _{k}{G}_{k}{e}^{i2\pi kx/D}(\begin{array}{cc}{e}^{ik\ell \theta } & 0\\ 0 & {e}^{-ik\ell \theta }\end{array})=\sum _{k}{G}_{k}{e}^{i2\pi kx/D}{\bf{W}}(k\ell \theta ).$$

Now, when this element is included in the Jones matrix sequence in Eq. (3), the QWPs transform these linear retarders into polarization rotators, so the Jones matrix **M** describing the grating can be written as:

7 $${\bf{M}}={\bf{H}}{\bf{W}}{\bf{P}}\cdot \sum _{k}{G}_{k}{e}^{i2\pi kx/D}{\bf{R}}(2kq\theta )=\sum _{k}{G}_{k}{e}^{i2\pi kx/D}{{\bf{M}}}_{kq}.$$

Note that each diffraction order *k* = ± 1, ±2, ±3 encodes a different *q*-plate **M**_*kq*_ with a charge given by the relation 2*q* = *k*ℓ. The phase grating is designed to provide the same intensity value of around |*G*_*k*_|^2^≈15% for all six target diffraction orders, thus giving equal strength for each encoded *q*-plate.

**Figure 2:**
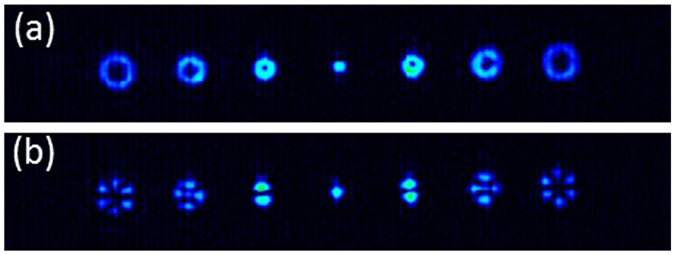
Experimental generation of 6 different vector beams. (a) Capture without analyzer; (b) Capture with vertical linear analyzer.

Figure 2 shows the corresponding experimental results. In Fig. 2(a) the image is captured without analyzer, thus not yet revealing the polarization structure of the focused beams. In this case six diffraction orders are generated, where the topological charge goes as *k*ℓ = ± 1, ±2, ±3, since the embedded spiral phases have values ℓ =  1. A zero-order beam is also produced, that appears as a bright spot denoting that no singularity appears in this order. The result looks like those in ref. 11, where scalar vortex beams where generated with a phase-only fork-grating, and shows doughnut beam focalizations with dark centers increasing with the absolute value of *k*. The grating design provides equal energy onto the six target diffraction orders. However, since higher diffraction orders with higher topological charges result in doughnut focalizations with larger diameter, the intensity becomes weaker as the order increases.

The vector beam nature of these beams is clearly revealed when an analyzer is included in the system. This is shown in Fig. 2(b), where a vertical linear analyzer is used. Now a number of 2*k*ℓ lobes appear in each diffraction order. These vector beams are produced by the encoded *q*-plate devices that the embedded spiral phases effectively generate in each diffraction order. In this case, since the input polarization is selected linear and vertical, the first *k* = 1 diffraction order reproduces the first order radial polarized beam. In addition, the other diffraction orders generate higher-order radial polarizations (this can be verified by the fact that all beams show a bright area at the vertical direction).

### Polarization grating design

Next we illustrate how to create the polarization grating. According to the procedure we described in ref. 17, we design now a vector grating by combining two different phase-only gratings, one affecting the horizontal linear polarization, and another affecting the vertical linear polarization.

Here, we selected a grating diffracting in the vertical direction, so each of the target diffraction orders in the grating is described by the linear phase term exp(*i*2*πky*/*D*), where *D* denotes again the period of the grating. Now each component is controlled by a different modulus and phase value, ${\mu }_{k}^{v}{e}^{i{\alpha }_{k}^{v}}$ for the vertical polarization, and ${\mu }_{k}^{h}{e}^{i{\alpha }_{k}^{h}}$ for the horizontal polarization, where super-indices *v* and *h* denote the gratings modulating the vertical and horizontal input polarization components respectively. Each grating provides the same set of diffraction orders *k*, but now different complex amplitudes ${G}_{k}^{v}$ and ${G}_{k}^{h}$ can be obtained for the vertical and horizontal polarizations. Therefore, the linear retarder Jones matrix **W** in Eq. (6) is now written as:

8 $${\bf{W}}=(\begin{array}{cc}\sum _{k}{G}_{k}^{v}{e}^{i2\pi ky/D} & 0\\ 0 & \sum _{k}{G}_{k}^{v}{e}^{i2\pi ky/D}\end{array}).$$

The parameters ${\mu }_{k}^{v},{\alpha }_{k}^{v},{\mu }_{k}^{h},{\alpha }_{k}^{h}$ are again numerically calculated to provide a desired constraint between the modulus and phase of the complex values ${G}_{k}^{v}=|{G}_{k}^{v}|{e}^{i{\beta }_{k}^{v}}$, ${G}_{k}^{h}=|{G}_{k}^{h}|{e}^{i{\beta }_{k}^{h}}$, at the diffraction orders generated by each grating. Note that for this case, it is crucial to impose restrictions on the modulus, but also on the phase values ${\beta }_{k}^{v},{\beta }_{k}^{h}$ in order to achieve polarization control at the diffraction orders.

The phase-only gratings in the vertical and horizontal polarization components are selected to provide the following complex values in the six target diffraction orders (*k* = + 3, +2, +1, −1, −2, −3):

9 $$|{G}_{k}^{v}|{e}^{i{\beta }_{k}^{v}}=(\frac{1}{\sqrt{2}}{e}^{i\pi },\frac{1}{\sqrt{2}},\frac{1}{\sqrt{2}}{e}^{i\pi /2},\frac{1}{\sqrt{2}},1,0).$$

10 $$|{G}_{k}^{h}|{e}^{i{\beta }_{k}^{h}}=(\frac{1}{\sqrt{2}}{e}^{-i3\pi /2},\frac{1}{\sqrt{2}},\frac{1}{\sqrt{2}}{e}^{-i\pi },\frac{1}{\sqrt{2}},0,1).$$

This way, the corresponding Jones matrices encoded onto each diffraction order are:

11 $$\begin{array}{c}{{\bf{W}}}_{+3}=\frac{{e}^{i\pi /4}}{\sqrt{2}}(\begin{array}{cc}{e}^{-i\pi /4} & 0\\ 0 & {e}^{+i\pi /4}\end{array});{{\bf{W}}}_{+2}=\frac{1}{\sqrt{2}}(\begin{array}{cc}1 & 0\\ 0 & 1\end{array});{{\bf{W}}}_{+1}=\frac{{e}^{i\pi /4}}{\sqrt{2}}(\begin{array}{cc}{e}^{+i\pi /4} & 0\\ 0 & {e}^{-i\pi /4}\end{array});\\ {{\bf{W}}}_{-1}=\frac{{e}^{i3\pi /4}}{\sqrt{2}}(\begin{array}{cc}{e}^{+i\pi /2} & 0\\ 0 & {e}^{-i\pi /2}\end{array});{{\bf{W}}}_{-2}=(\begin{array}{cc}1 & 0\\ 0 & 0\end{array});{{\bf{W}}}_{-3}=(\begin{array}{cc}0 & 0\\ 0 & 1\end{array}).\end{array}$$

We again follow the convention defined in ref. 30. Note that the orders *k* = + 3, +2, +1 and −1 encode linear retarders, with retardances of –*π*/2, 0, +*π*/2 and *π* respectively, while orders *k* = −2 and *k* = −3 encode vertical and horizontal linear polarizers respectively. Therefore, when placed in between QWP1 and QWP3, the polarization element encoded onto diffraction orders *k* = + 3, +2, +1 and −1 are polarization rotators (see Equation (3)) of rotation angles of –*π*/4, 0, +*π*/4 and *π*/2 respectively. Finally the diffraction orders *k* = −2 and *k* = −3 encode LCP and RCP polarizers. Again, we used the procedures outlined in ref. 14–16 to optimize this grating. We note that we have 10 variables to optimize (the 5 amplitudes and 5 phases for each grating).

**Figure 3:**
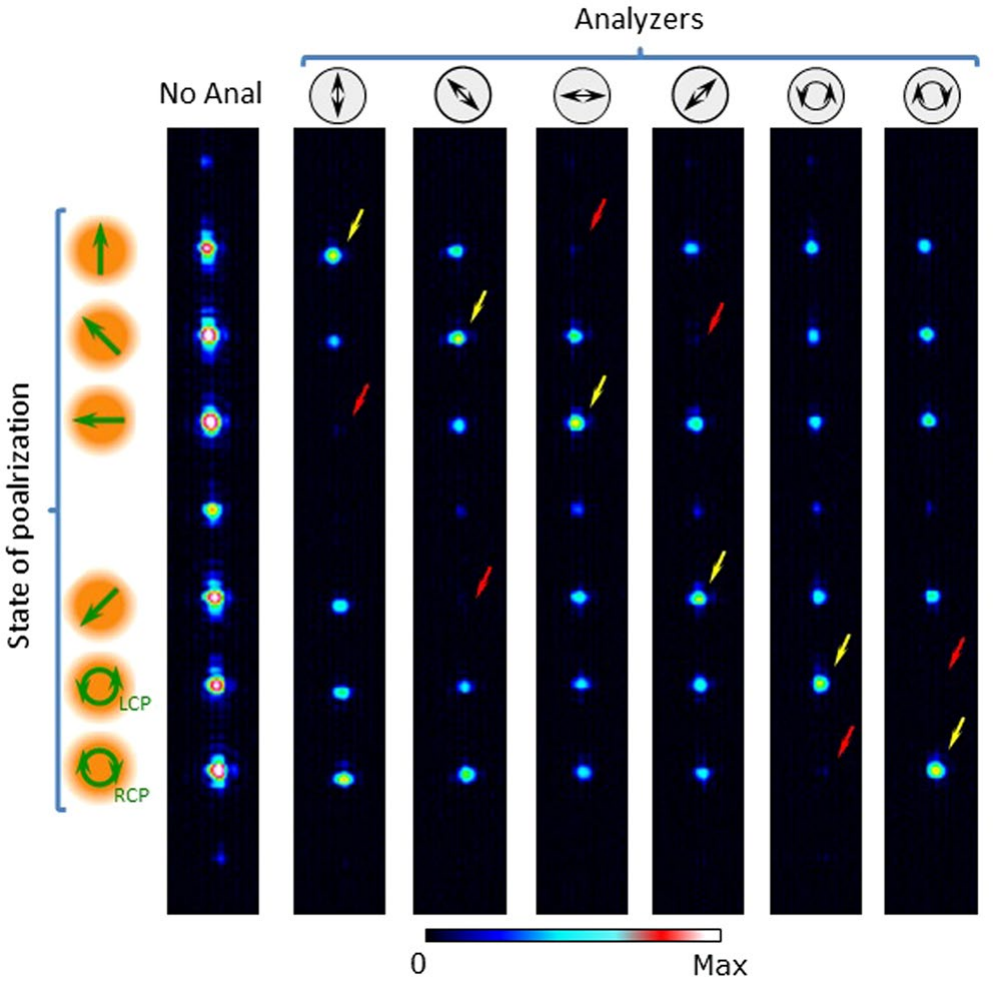
Experimental generation of a polarization diffraction grating generating 6 orders with different states of polarization. “No Anal” indicates the result without analyzer. The symbol on top indicates the analyzer used in the other cases. The yellow arrow points the bright spot indicating the order with the state of polarization coincident with the analyzer, while the red arrow points the null diffraction order corresponding to the orthogonal state.

Figure 3 shows the corresponding experimental results, similar to those presented in ref. 29, but using a slightly different design in order to consider the above-mentioned action of QWP1 and QWP3 in the system in Fig. 1. The polarization diffraction grating is selected to diffract in the vertical direction. Here the input polarization is selected to be linearly polarized oriented at 45°. Six diffraction orders with equal intensity are generated, but the states of polarization change in each order, according to the definition in the left column of Fig. 3. In the diffraction order *k* = + 3, the polarization will be rotated by −45°, to become vertically polarized. In order *k* = + 2 the polarization will remain unchanged. In orders *k* = + 1 and *k* = −1, the polarizations will be rotated by +45° and +90° respectively. Finally, in orders *k* = −2 and *k* = −3, the polarizations will be converted into LCP and RCP.

The experimental results in Fig. 3 confirm this design. The column named “No Anal” refers to the case when no analyzer is placed before the detector, and shows the effective generation of the six target diffraction orders *k* = ± 1, ±2, ±3. Although the total diffraction efficiencies of the phase gratings reach about 90%, some other diffraction orders are visible because we captured saturated images. The images are saturated in order to show that, when a polarization analyzer is included in the system before the CCD detector, the diffraction order with the orthogonal state of polarization vanishes, as shown in Fig. 3 marked with a red arrow. On the other hand, the yellow arrow marks the position of the order with the same polarization as transmitted by the analyzer. These results show the ability to generate six different polarization states in parallel at the six different vertically diffracted target orders.

## Results: Building a Multiple Vector Beam Generator

**Figure 4:**
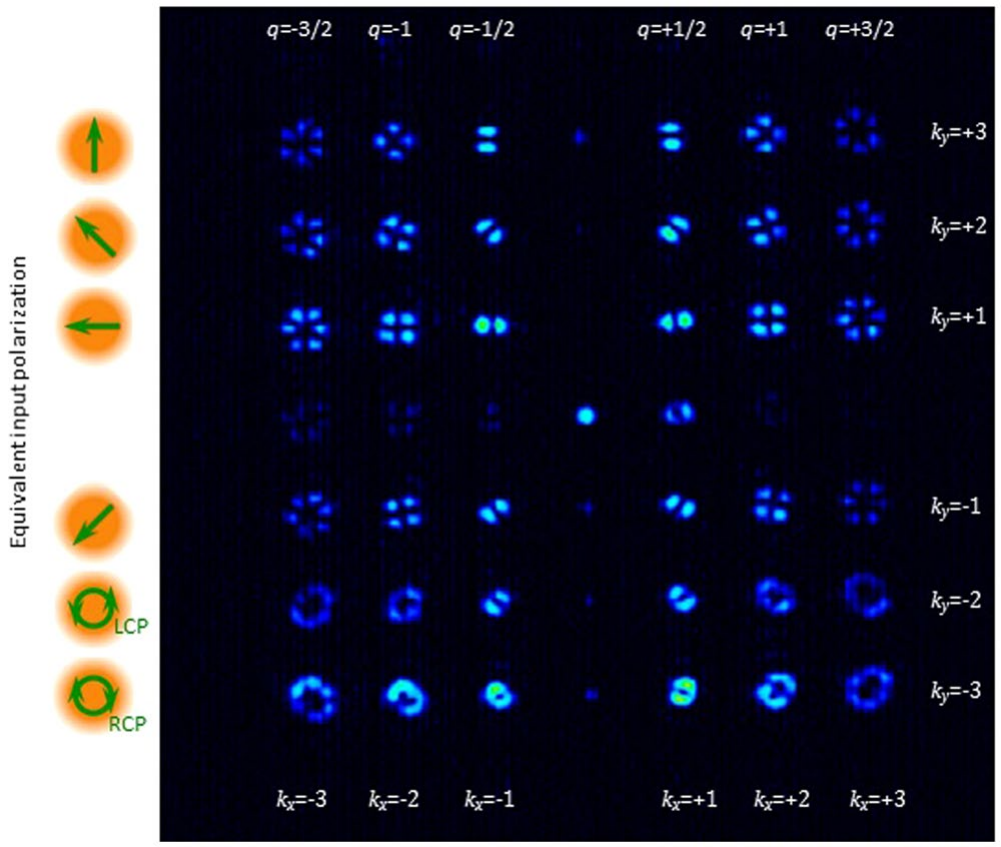
Experimental single-shot generation of 6 × 6 vector beams. Horizontal orders encode *q*-plates with different *q*-values, while vertical orders have different states of polarization.

In this section we combine the two gratings presented in the previous subsections: we combine the multiple *q*-plate grating diffracting in the horizontal direction, with the polarization grating diffracting in the vertical direction. As a consequence, we can generate an array of 6 × 6 diffraction orders. The inset in Fig. 1 shows the two phase masks that are addressed to each side of the SLM. We note that these two circular phase masks have a radius of only 120 pixels and require precise alignment to superimpose the pixels for each grating. In spite of this limited resolution, the experimental results in Fig. 4 demonstrate the successful generation of an array of 6 × 6 vector beams.

Again, the system is illuminated with a collimated beam with uniform linear polarization oriented at 45°. The two dimensional grating generates an array of 6 × 6 diffraction orders, each one with a different vector beam. The final analyzer is linear and vertically oriented. The horizontal order *k*_*x*_ encodes different *q*-plate devices, with *q*-values *q* = *k*_*x*_/2, while the vertical orders *k*_*y*_ encode different equivalent input polarizations corresponding to the six cardinal points of the Poincaré sphere, with the same sequence as in Fig. 3. As a result, we generate the 36 vector beams, generated with encoded *q*-plate devices with values *q* = ± 1/2, *q* = ± 1, and *q* = ± 3/2 (along orders diffracted horizontally), each one as illuminated with the six input cardinal polarizations (along orders diffracted vertically). Note that a given *q*-plate generates different output vector beams of the same charge for different input polarizations^25^.

Therefore, this pattern in Fig. 4 shows the single-shot parallel generation of all six cardinal vector beams of the higher order Poincaré spheres of topological charges ℓ = ± 1, ℓ = ± 2, and ℓ = ± 3. The difference in charge can be noticed in the size of the singularity and in the number of bright lobes (for linear states) that appear in each diffraction order. The difference in the sign of the charge can be noticed in the opposite sense that the lobes rotate with the equivalent input polarization (see for instance in the second row, the first 3 patterns rotate counterclockwise relative to the first row while the next 3 patterns rotate in the clockwise direction.

Next, we discuss how to use these gratings as a vector beam polarization spectrum analyzer where we can detect both the charge of the vector beam and the polarization state onto which it is imprinted.

### Vector Beam Polarization Spectrum Analyzer

The same diffraction grating demonstrated above can be used to detect an input vector beam in a similar manner as a vortex diffraction grating can detect a scalar vortex beam^11^. If the beam incident on the system is already a vector beam, the spiral phase structures in the incoming beam cancel those phases embedded in the gratings, and the result is a delta function at a higher diffraction order where these phases cancel^33^. If the incoming vector beam is generated with another *q*-plate, the phenomena can be regarded as a subtraction of the generating and detecting *q*-plate^34^.

### Detection of the vector beam order

In order to explore this idea, it is interesting to examine the matrix representation for the *q*-plates and how we can combine two *q*-plates to subtract their values.

Consider a vector beam illuminating the system in Fig. 1. This vector beam can be generated by illuminating a *q*-plate with charge ℓ_*in*_ = 2*q*_*in*_ with a given homogeneous state of polarization *E*_0_. For simplicity, let us first consider the one dimensional *q*-plate grating previously demonstrated, and described with Equation (7). The complete sequence of a vector beam generated with an input *q*-plate, launched onto the system in Fig. 1 can be described as:

12 $${E}_{out}=(\sum _{k}{G}_{k}{e}^{i2\pi kx/D}{{\bf{M}}}_{kq})\cdot ({{\bf{M}}}_{{q}_{in}}{E}_{0}),$$

where ${{\bf{M}}}_{{q}_{in}}{E}_{0}$ describes the vector beam input to the system, and *E*_*out*_ denotes the output of the system, that will consist of an array of different vector beams diffracted on the horizontal direction.

Considering the Jones matrices for a *q*-plate, it is direct to show that the multiplication of two *q*-plates matrices follows ${{\bf{M}}}_{{q}_{2}}{{\bf{M}}}_{{q}_{1}}={\bf{H}}{\bf{W}}{\bf{P}}\cdot {{\bf{M}}}_{{q}_{1}-{q}_{2}}$ ^34^, so the above equation can be rewritten as:

13 $${E}_{out}={\bf{H}}{\bf{W}}{\bf{P}}\cdot (\sum _{k}{G}_{k}{e}^{i2\pi kx/D}{{\bf{M}}}_{{q}_{in}-kq})\cdot {E}_{0},$$

Therefore, note that when the order of the *q*-plate generating the input vector beam matches the order of one of the *q*-plates encoded in the grating, so *q*_*in*_−*kq* = 0, then the matrix ${{\bf{M}}}_{{q}_{in}-kq}$ becomes an identity matrix and the output is no longer a vector beam but a uniformly polarized beam. Also note that, since we are not incorporating the final HWP in the system in Fig. 1, the final HWP term in Eq. (13) can be omitted and the polarization of the uniformly polarized output beam obtained when the charges match is defined simply by the input state *E*_0_.

**Figure 5:**
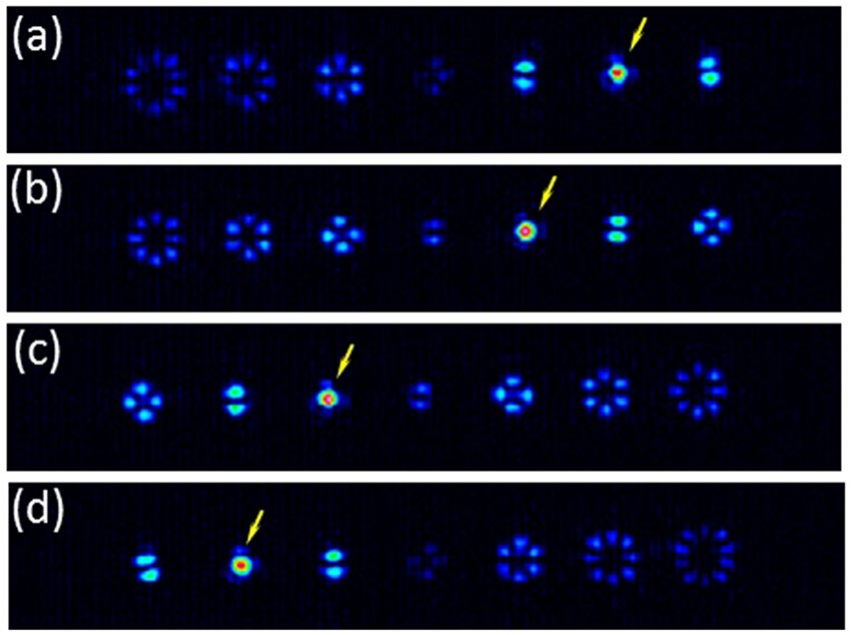
Experimental detection of input vector beam. A vertical linear analyzer is used in all cases. The input beam is generated with vertical linearly polarized light passing through a *q*-plate device with: (**a**) *q* = + 1, (**b**) *q* = + 1/2, (**c**) *q* = −1/2, (**d**) *q* = −1. The yellow arrow points the bright spot indicating the detection of a vector beam.

In order to probe this idea, Fig. 5 shows the captured patterns when the same grating producing the pattern in Fig. 2 is now illuminated with a vector beam generated with a *q*-plate. We used two commercial *q*-plates from Thorlabs with *q*-values *q* = 1/2 and *q* = 1 respectively, thus generating input vector beams with topological charges ℓ = 1 and ℓ = 2 respectively. The *q*-plates were placed in the input beam before entering the system in Fig. 1. The initial polarization was selected now linear and vertical. Therefore, the higher order radial polarizations are input beams to the system. The final analyzer before the CCD detector is selected linear and vertical. The resulting patterns in Fig. 5(a) and (b) show how the diffraction order showing the bright spot has now moved to the second and first diffraction orders when input *q*-plates with *q* = 1 and *q* = 1/2 are selected respectively. Note how the rest of the diffraction orders show vector beams, but the charge is symmetric around the order showing the delta function (bright spot).

In Fig. 5(c) and (d) the same results are obtained but now with an input vector beam with negative charge. This is accomplished by inserting the *q*-plate in between two half-wave plates, what results in an equivalent *q*-plate with negative value^34^. Therefore, we generate *q*-plates with *q* = −1/2 and *q* = −1. Now we see how the bright spot shifts to the left orders, providing a positive detection of the corresponding vector beams.

These results thus show a generalization to vector beams of the technique previously used with scalar vortex beams^11^, which is now widely used as a mode sorter in vortex based communication systems^4, 9^.

### Polarimetric detection of vector beams

Finally, in this last subsection we probe the use of the two-dimensional grating in Section 3.2 as a vector beam sorter element, where both the topological charge and the state of polarization can be simultaneously determined in a single shot. To discuss this subject, we analyze how we can detect a given vector beam.

Note that the Jones matrix sequence can be written as:

14 $${E}_{out}=(\sum _{{k}_{y}}{G}_{k}{e}^{i2\pi {k}_{y}y/D}{{\bf{M}}}_{{k}_{y}})\cdot (\sum _{{k}_{x}}{G}_{k}{e}^{i2\pi {k}_{x}x/D}{{\bf{M}}}_{{k}_{x}q})\cdot ({{\bf{M}}}_{{q}_{in}}{E}_{0}),$$

Now we have a two dimensional grating, where the horizontal direction encodes *q*-plate devices ${{\bf{M}}}_{{k}_{x}q}$ that depend on the horizontal order *k*_*x*_ while the vertical direction encodes the different polarization elements ${{\bf{M}}}_{{k}_{y}}$ described in subsection 3.2, i.e., orders *k*_*y*_ = + 3, +2, +1 and −1 encode polarization rotators of rotation angles of −*π*/4, 0, +*π*/4 and *π*/2 respectively, and orders *k*_*y*_ = −2 and *k*_*y*_ = −3 encode LCP and RCP polarizers.

Therefore, again, when the encoded *q*-plate matches the order with the incoming vector beam, a uniformly polarized beam is produced (a delta function diffraction order), and the state of polarization will be given by the product ${{\bf{M}}}_{{k}_{y}}{E}_{0}$. Since the six polarization elements encoded in the vertical direction provide six transformations yielding the six cardinal states of the Poincaré sphere out of input vertical polarized state, the system can be used to perform generalized polarimetry measurements on vector beams.

**Figure 6:**
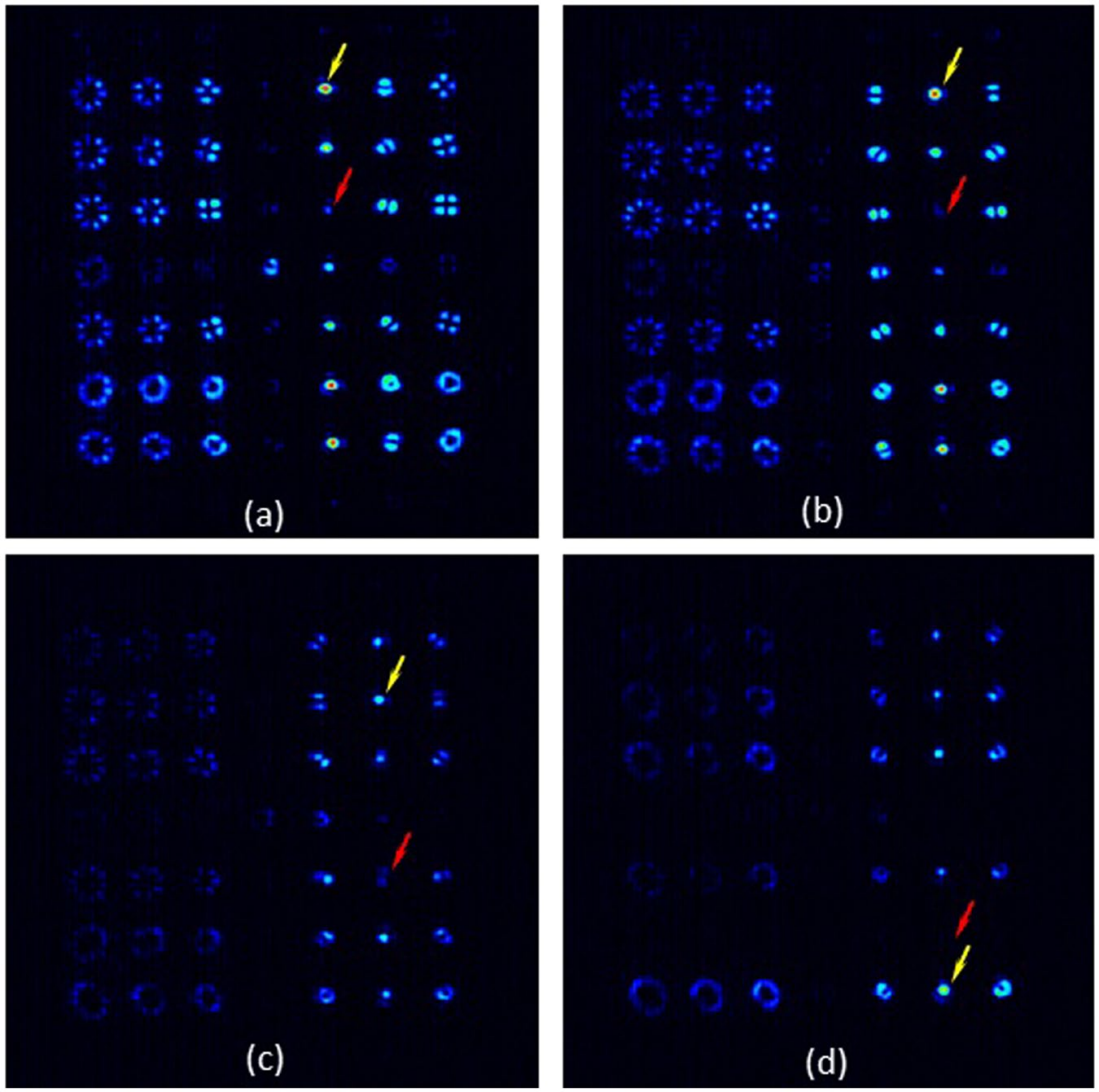
Experimental detection of vector beams generated with an external *q*-plate illuminated with different polarizations: (**a**) Input *q* = 1/2 plate illuminated with linear vertical polarization, (**b**) Input *q* = 1 plate illuminated with linear vertical polarization, (**c**) Input *q* = 1 plate illuminated with linear polarization oriented at 45° and (**d**) Input *q* = 1 plate illuminated with RCP polarization. The yellow arrow points the bright spot indicating the detection of a vector beam, while the red arrow points the null diffraction order corresponding to the orthogonal state.

The experimental results are shown in Fig. 6. For that purpose, again we use the commercial *q*-plates to generate different input vector beams that are then launched to the system in Fig. 1 when displaying the grating generating the 6 × 6 pattern in Fig. 5. The results shown in Fig. 6 correspond to four different input beams. In all cases we obtain again an array of 6 × 6 beams as in Fig. 5, but notice that in each case there is a diffraction order that shows a bright point (delta function) revealing the detection of a given vector beam. This order is indicated with a yellow arrow, while the red arrow indicates the position of the diffraction order corresponding to the orthogonal polarization state, which shows a null intensity.

In Fig. 6(a) the input beam is generated using the input *q*-plate with *q* = 1/2 illuminated with vertical linear polarization. Thus, the beam impinging the system is the radially polarized beam. Note how an intense bright diffraction order appears on the (*k*_*x*_ = + 1, *k*_*y*_ = + 3) location, while the order at location (*k*_*x*_ = + 1, *k*_*y*_ = + 1) vanishes (this order corresponds to the azimuthal polarization, which is the vector beam orthogonal to the radial polarization).

In Fig. 6(b) the input *q*-plate has *q* = 1, and again the illumination is with linear vertical polarization. Thus, the second order radial polarization is generated as the input beam. Note how now the delta function has shifted to (*k*_*x*_ = + 2, *k*_*y*_ = + 3) and the null order has shifted to (*k*_*x*_ = + 2, *k*_*y*_ = + 1).

Finally, Fig. 6(c) and (d) show the results when the same input *q* = 1 plate is illuminated with linearly polarized light oriented at 45°, and with RCP polarization respectively. Therefore, in the first case a slanted second order vector beam is generated (Fig. 6(c)), while in the second case a scalar LCP vortex beam is generated (Fig. 6(d)) as the beam input to the vector beam polarization state spectrum analyzer. Note that in these two cases the bright spot keeps the horizontal order *k*_*x*_ = + 2, as corresponds to a vector beam with topological charge 2, but appears at different vertical orders, corresponding to the different polarization state.

Note that, in fact, the relative intensities of the six different delta functions appearing along the vertical direction corresponding to the detected topological charge are a direct measurement of the generalized Stokes parameters of the input vector beam. Thus the vector beam spectrum analyzer allows performing single shot multiple higher-order polarimetric measurements. However, as we said earlier, this is a proof of concept experiment. Our detector array does not have sufficient resolution to accurately measure the strengths of the delta functions. Such precise higher-order polarimetric measurements would require better resolution both in the detector, and in the SLM encoding the grating.

## Discussion

These results demonstrate the experimental realization of a vector beam polarization diffraction grating capable to generate, in a single shot, an array of vector beams with different topological charges and different states of polarization. The same system can be used as a vector beam polarization state spectrum analyzer, capable to identify the order of an input vector beam as well as the particular state of polarization.

This special polarization diffraction grating has been experimentally realized with an optical system using a single parallel-aligned SLM, and the experimental results probe in each case the successful parallel generation of an array of vector beams, or the successful detection of the order and particular polarization of an input vector beam.

This kind of approach generalizes to vector beams previous techniques for scalar vortex beam detection, now widely used in orbital angular momentum based communication systems. Therefore, we believe this generalization might find relevant applications in such systems, where channels with different topological change and channels with a different state of polarization can be generated/detected in parallel. In addition, we note that the detection scheme can also be described as a vector beam polarization correlation system. This approach therefore generalizes to vector beams the mode sensing technique that has been used for years in multichannel correlators^35^ and in modal wavefront sensors^36^, and recently also for the orbital angular momentum density of light^37^.

Our emphasis in this experiment involves the use of these vector beams in optical communication applications where each vector beam is projected onto the 6 principal points on the appropriate Poincaré sphere. However, there are several different applications. First, we have demonstrated complete control over the polarization states for each order. Obtaining any elliptically polarized state requires the design of the appropriate gratings as discussed earlier. In addition, we have the components necessary for building the Stokes parameters if they are required.

We have two final observations. First, we have not utilized the programmability of the SLM. Instead, we concentrate on generating multiple output channels in parallel. Obviously, the capability to program the SLM adds an additional element. For higher efficiency and resolution in applications requiring more elements, one would probably design fixed photolithographic polarizing optical elements to obtain similar results with lower costs.
